# Metformin Inhibits Cell Motility and Proliferation of Triple-Negative Breast Cancer Cells by Blocking HMGB1/RAGE Signaling

**DOI:** 10.3390/cells14080590

**Published:** 2025-04-13

**Authors:** Shazie Yusein-Myashkova, Desislava Vladimirova, Anastas Gospodinov, Iva Ugrinova, Jordana Todorova

**Affiliations:** Roumen Tsanev Institute of Molecular Biology, Bulgarian Academy of Sciences, Acad G. Bonchev Str., Bl. 21, 1113 Sofia, Bulgaria; shazi@abv.bg (S.Y.-M.); desivladimirova@abv.bg (D.V.); agg@bio21.bas.bg (A.G.); ugryiva@gmail.com (I.U.)

**Keywords:** HMGB1, breast cancer, metformin, metastasis, EMT

## Abstract

High-mobility group box 1 (HMGB1) is a nuclear chromatin protein overexpressed in various cancers and linked to tumor progression. Outside the cell, HMGB1 binds to receptors such as the receptor for advanced glycation end products (RAGE), promoting metastasis. Targeting this signaling pathway may provide a new therapeutic strategy for aggressive cancers. Metformin, a well-established antidiabetic drug, directly interacts with HMGB1, inhibiting its pro-inflammatory functions. This study investigates metformin’s effects on the HMGB1/RAGE signaling pathway in triple-negative breast cancer (TNBC) cells. Using wound-healing and colony formation assays, we demonstrate that metformin reduces HMGB1-induced cell migration and proliferation. Immunoblotting and immunofluorescence analyses reveal that metformin decreases RAGE stabilization on the cell membrane, disrupts NF-κB signaling, and reverses the epithelial-to-mesenchymal transition (EMT) by increasing E-cadherin, reducing vimentin, and stabilizing β-catenin at the cell membrane. Furthermore, metformin lowers HMGB1 and RAGE protein levels, disrupting the positive feedback loop that promotes cancer aggressiveness. These findings highlight metformin’s potential as a therapeutic agent in TNBC by inhibiting HMGB1/RAGE-driven metastasis.

## 1. Introduction

Breast cancer remains the most common cancer worldwide in women, with high incidence, accounting for approximately 15% of all diagnosed cases per year in the USA [1]. Triple-negative breast cancer (TNBC) is an aggressive and heterogeneous subtype of breast cancer that lacks the expression of estrogen receptor (ER), progesterone receptor (PR), and human epidermal growth factor receptor 2 (HER2). Due to the absence of these molecular targets, TNBC remains challenging to treat, with limited therapeutic options and a high risk of metastasis. Recent studies suggest that dysregulation of inflammatory and metabolic pathways is critical in TNBC progression, identifying potential new targets for therapeutic intervention [2]. One such pathway involves the high-mobility group box 1 (HMGB1) protein and its receptor for advanced glycation end products (RAGE). HMGB1 is a multifunctional protein with important nuclear and extracellular functions [3]. The protein is also pivotal in tumor progression as it promotes inflammation, cell migration, and survival [4]. Clinical studies have shown that patients with metastatic breast cancer exhibit significantly higher concentrations of HMGB1 in their blood serum compared to healthy controls [5]. In addition, it has been shown that high serum levels of HMGB1 or its expression at tumor sites correlate with poor prognosis and therapy outcomes [6]. This suggests that HMGB1 may contribute to TNBC aggressiveness by promoting tumor progression and resistance to treatment.

Extracellular HMGB1 primarily interacts with RAGE [7] and Toll-like receptors (TLR2, TLR4 [8], and TLR5) [9]. NF-κB is a key transcription factor that links inflammation with cancer and is a downstream target of HMGB1/RAGE signaling [10]. This signaling promotes tumor migration and invasion in liver cancer [11], cervical cancer [12], prostate cancer [13], and pancreatic cancer [14]. Co-expression of HMGB1 and RAGE was also associated with the enhanced invasive and metastatic potential of esophageal squamous cell carcinoma [15], prostate cancer [16], and renal cell carcinoma [17]. Silencing of RAGE reduces tumor growth [18]. Blocking HMGB1 and its receptor RAGE by using specific inhibitors resulted in similar outcomes [19], suggesting that the interaction between HMGB1 and RAGE is a crucial driver of cancer progression. Since TNBC lacks targeted therapies, HMGB1/RAGE inhibition is a promising new research direction. Recently, a RAGE-binding peptide [20] demonstrated a promising inhibitory effect on cancer growth, along with other small-molecule RAGE inhibitors [21] showing potential in suppressing metastasis in triple-negative breast cancer.

Metformin is the most widely prescribed anti-hyperglycemic agent for type 2 diabetes worldwide. Beyond its anti-hyperglycemic effect, metformin also has anti-oxidant [22] and anti-inflammatory [23] properties. Metformin lowers the risk of breast, colon, and prostate cancer in people with type 2 diabetes [24]. The drug inhibits cellular proliferation in patients with endometrial cancer [25] and prostate cancer [26]. Horiuchi et al. demonstrated that metformin binds directly to HMGB1 and inhibits its pro-inflammatory activity [27]. Other studies showed that the drug blocks HMGB1 release in rabbit annulus fibrosus progenitor cells [28] and RAW 264.7 cells [29]. Metformin has also been shown to decrease HMGB1 serum levels in patients with rheumatoid arthritis [30]. While there is research on metformin’s general anticancer effects and its influence on inflammatory pathways, direct investigations into its specific modulation of the HMGB1/RAGE axis in TNBC have not been conducted.

This study aimed to elucidate the role of metformin in modulating the HMGB1/RAGE pathway in TNBC. We investigated metformin’s effects on RAGE stabilization at the cell membrane, NF-κB activation, MMP-2 expression, EMT markers, and TNBC cell motility and proliferation. Our findings provide new insights into metformin’s potential as a therapeutic agent in TNBC by demonstrating its ability to disrupt HMGB1-induced oncogenic signaling.

## 2. Materials and Methods

### 2.1. Cell Culture

The human breast cancer cell lines MDA-MB-231 and MDA-MB-468 (TNBC cell lines) were acquired from the American Type Culture Collection (ATCC, Manassas, VA, USA). MDA-MB-231 cells were cultivated in DMEM, high glucose (Cat# 11965092, Gibco™, Thermo Fisher Scientific, Waltham, MA, USA) media, while MDA-MB-468 cells were grown in RPMI 1640 (Cat# 11875093, Gibco™, Thermo Fisher Scientific, Waltham, MA, USA) media. Both media were supplemented with 10% FBS (Fetal Bovine Serum, certified (Cat# 16000044, Gibco™, Thermo Fisher Scientific, Waltham, MA, USA) (Gibco, USA) and 1% antibiotics (Antibiotic Antimycotic Solution (100×), Stabilized (Cat# A5955, Sigma-Aldrich, St. Louis, MO, USA). The cells were grown at a controlled temperature of 37 °C with 5% CO_2_. The cells used for the experiments were between 2 and 8 passages. DAPI staining did not show any sign of Mycoplasma contamination.

### 2.2. MTT Colorimetric Assay

Three thousand cells were seeded in 96-well plates and treated 24 h later with different doses of metformin. After 72 h, MTT (3-(4,5-Dimethylthiazol-2-yl)-2,5-Diphenyltetrazolium Bromide) (Cat# M6494, Invitrogen™, Thermo Fisher Scientific, Waltham, MA, USA) solution (0.5 mg/mL in PBS) was added to each well of the culture plate. A further 3 h of incubation allowed the formation of purple insoluble formazan crystals. The latter crystals were dissolved in DMSO (Cat# D2438, Sigma-Aldrich, St. Louis, MO, USA) and absorbance was read at 550 nm. The collected absorbance values were blanked against MTT and DMSO and normalized to the mean value of the untreated control (100% cell viability) [31].

### 2.3. Preparation of Plasmid Constructs and Purification of Recombinant Hmgb1 Proteins

The full-length form of HMGB1 protein cloned in pET28a+ plasmid (pET-28a(+) DNA (Cat# 69864-3, Novagen®, MilliporeSigma, Burlington, MA, USA) was expressed in modified *Escherichia coli* BL21 Poly Lys S as previously described [32]. The recombinant protein was purified using His-tag resin (HIS-Select® Nickel Affinity Gel (Cat# P6611, Millipore, MilliporeSigma, Burlington, MA, USA) according to the manufacturer’s protocol.

### 2.4. Western Blot Analysis

For total protein lysates, the cells were washed in 1× PBS (Cat# P5493, Sigma-Aldrich, St. Louis, MO, USA) and lysed in 1× SDS sample buffer (50 mM Tris HCl (pH 6.8), 2% SDS, 10% glycerol, 1% β-mercaptoethanol, 12.5 mM EDTA, and 0.02% bromophenol blue) for 10 min, followed by 3–5 s of sonication. For the nuclear protein lysates, cells were harvested by trypsinization and transferred into 1.5 mL tubes in hypotonic lysis buffer in the presence of cocktail protease inhibitors. After passing the cells through a syringe needle 10 times until all cells were lysed, the cell lysate was left for 30 min on ice. After 5 min of centrifugation at 720× *g* (3000 rpm), the supernatant containing the cytoplasmic cell elements was removed. Pellets containing the nuclei were resuspended in 1× TBS with 0.1% SDS or 0.5% NP40. After that, the suspensions were vortexed briefly and then sonicated for 5 s to shear genomic DNA and homogenize the lysate. All centrifugations were performed at 4 °C. The samples were kept on ice throughout the whole procedure [33]. Finally, the SDS sample buffer was added to a final concentration of 1×. The protein samples were boiled for 5 min at 96 °C, separated by 8 or 12% SDS-PAGE, and then transferred onto a nitrocellulose membrane. Subsequently, the membrane was blocked with 5% bovine serum albumin in Tris-buffered saline containing Tween (TBST: 50 mmol/L Tris-HCl, 150 mmol/L NaCl, and 0.1% Tween) for 1 h at room temperature. The membranes were incubated with primary antibodies overnight at 4 °C in appropriate dilutions. The following antibodies were used: Anti-RAGE antibody (Cat# ab216329, Abcam, Cambridge, MA, USA) 1:1000; Anti-HMGB1 antibody (Cat# ab18256, Abcam, Cambridge, MA, USA) 1:1000; Anti-Vimentin (D21H3) XP® Rabbit mAb (Cat# 5741, Cell Signaling Technology, Danvers, MA, USA) 1:1000; Anti-β-Catenin Antibody (Cat# 9562, Cell Signaling Technology, Danvers, MA, USA) 1:1000; Anti-NF-κB p65 (D14E12) XP® Rabbit mAb (Cat# 8242, Cell Signaling Technology, Danvers, MA, USA) 1:500; Anti-E-Cadherin (4A2) Mouse mAb (Cat# 14472, Cell Signaling Technology, Danvers, MA, USA) 1:1000; Purified anti-human/mouse/rat PCNA (BioLegend, San Diego, CA, USA) 1:500; Anti-β-Actin (ACTB) Antibody (Cat# A2228, Sigma-Aldrich, St. Louis, MO, USA) 1:2000. The membranes were washed three times with TBST and were then incubated with Goat anti-Mouse IgG (H+L) Secondary Antibody, HRP (Cat# 31430, Invitrogen™, Thermo Fisher Scientific, Waltham, MA, USA) (1:10,000) or Goat anti-Rabbit IgG (H+L) Secondary Antibody, HRP (Cat# 31460, Invitrogen™, Thermo Fisher Scientific, Waltham, MA, USA) (1:10,000) secondary antibodies for 1 h at room temperature. Following additional washes, the resulting protein bands were visualized using an enhanced chemiluminescence Western blotting detection system (Super Signal West PICO Plus Chemiluminescence Substrate (Thermo Fisher Scientific, Inc., Waltham, MA, USA), following the manufacturer’s instructions. β-actin was used as an internal reference, and the intensity of the protein band was quantified using ImageJ software (bundled with 64-bit Java 8, National Institutes of Health, Bethesda, MD, USA; https://imagej.nih.gov/ij/) [34].

### 2.5. Wound-Healing Assay

MDA-MB-231 and MDA-MB-468 were seeded in 12-well plates and allowed to grow to 100% confluency. A straight scratch was made using a sterile pipette tip, and the cells were rinsed with 1× PBS to remove cellular debris. To investigate the effect of HMGB1/RAGE signaling on cell motility, the cells were treated with 800 ng/mL recombinant HMGB1 protein in fresh media with reduced levels of FBS (0.1% for MDA-MB-321 and 1% for MDA-MB-468). For the experiment on metformin’s influence on HMGB1/RAGE signaling, the cells were treated with 1 mM (for MDA-MB-468 cells) or 3 mM (for MDA-MB-231 cells) metformin. Phase-contrast microscope images of the scratched areas were captured Zeiss AxioVert 200 M (Zeiss, Oberkochen, Germany) immediately after the scratch (0 h) and after 24 and 48 h. The extent of cell migration into the scratched area was evaluated using ImageJ software, and the closure percentage of the scratch was measured to assess migration potential [35].

### 2.6. Immunofluorescence

Cells were seeded onto glass coverslips in 24-well plates. The next day, they were treated with 800 ng/mL HMGB1 or 1 mM metformin (for MDA-MB-468 cells) or 3 mM metformin (MDA-MB-231 cells) for 24 h. The samples were washed with PBS, fixed in 4% paraformaldehyde for 20 min, and then permeabilized with 0.1% Triton X-100 (Cat# T9284, Sigma-Aldrich, St. Louis, MO, USA) in PBS. Then, the cells were incubated with blocking buffer (10% fetal calf serum, 1% BSA, and 0.1% Triton X-100 in PBS) for 1 h. After that, the coverslips were incubated with the primary antibodies at 4 °C overnight. The following antibody dilutions were used: Anti-RAGE antibody (Cat# ab216329, Abcam, Cambridge, MA, USA) 1:400; Anti-Vimentin (D21H3) XP® Rabbit mAb (Cat# 5741, Cell Signaling Technology, Danvers, MA, USA) 1:200; Anti-β-Catenin Antibody (Cat# 9562, Cell Signaling Technology, Danvers, MA, USA) 1:200; Anti-NF-κB p65 (D14E12) XP® Rabbit mAb (Cat# 8242, Cell Signaling Technology, Danvers, MA, USA) 1:200; and MMP-2 (D4M2N) Rabbit mAb (Cat# 40994, Cell Signaling Technology, Danvers, MA, USA) 1:500. After washing with PBS, the coverslips were incubated with secondary Alexa Fluor 488 Polyclonal Antibody (Cat# A-11094, Invitrogen™, Thermo Fisher Scientific, Waltham, MA, USA) for 1 h at 37 °C. After two washes in PBS, the coverslips were rinsed in ddH2O and briefly dipped in 100% EtOH. After a quick dry, the coverslips were mounted on a slide with Fluoromount-G™ Mounting Medium (Cat# 00-4958-02, Invitrogen™, Thermo Fisher Scientific, Waltham, MA, USA) containing 400 ng/mL DAPI. Images were taken using an epifluorescence microscope Zeiss AxioVert 200 M (Zeiss, Oberkochen, Germany) using a 63× objective.

### 2.7. EsiRNA Preparation and RNA Interference

Endoribonuclease-prepared siRNAs (esiRNAs) specifically targeting the coding regions of RAGE were synthesized using an endoribonuclease preparation method, as previously described in [36,37]. Primers for targeting the specific regions of RAGE were selected from the Riddle database [38]. The primer sequences used were as follows: forward primer 5′-TCACTATAGGGAGAGGATCCAGGATGAGGGGATTT and reverse primer 5′-TCACTATAGGGAGACGCTACTGCTCCACCTTCTGG. For the RNA interference experiment, each well of a 24-well plate with a transfection volume of 500 µL contained 30 pmol of esiRNA and 2 µL of Lipofectamine 2000. The knockdown efficiency of RAGE was assessed using Western blotting analysis.

### 2.8. Colony Formation Assay

A total of 500 cells of the MDA-MB-231 cell line were seeded into each well of 6-well plates. The next day, the medium was replaced with a new medium supplemented with 1.7 mM metformin or 800 ng/mL recombinant HMGB1 or both. The cells were left to grow for 8 days. Then, they were fixed in 4% paraformaldehyde for 10 min and stained with 0.1% crystal violet for 15 min [39]. Finally, the plates were imaged, and the number of colonies was counted using Image J software.

### 2.9. Statistical Analysis

The data are presented as the mean values ± SD of three independent experiments. One-way ANOVA (Graph Pad Prism8, San Diego, CA, USA) was used to compare the means between groups. *p*-values < 0.05 were considered to indicate statistically significant results.

## 3. Results

### 3.1. Metformin Inhibits RAGE Receptor Stabilization on the Cellular Membrane and Downstream HMGB1/RAGE Signaling

The binding of ligands to the RAGE receptor promotes oligomer stabilization and enhances cell signaling, regulating key cellular processes such as migration, proliferation, and adhesion [40]. We hypothesize that metformin binding to HMGB1 may disrupt the HMGB1/RAGE interaction, affecting RAGE stability on the cellular membrane.

**Figure 1:**
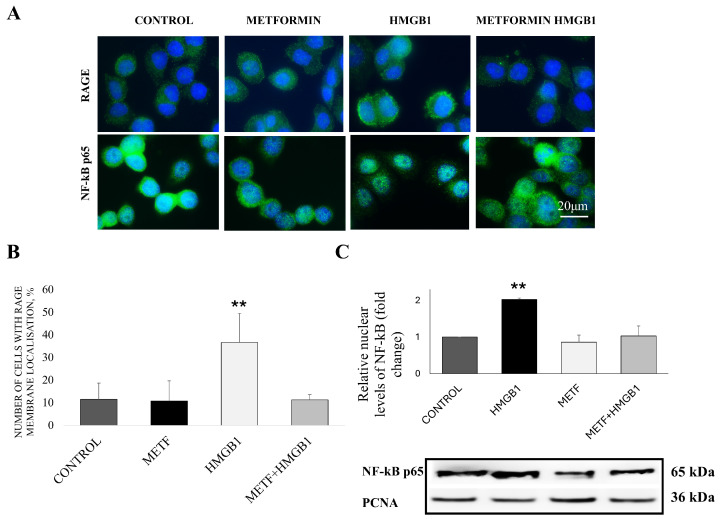
Effect of metformin on RAGE and NF-kB p65 protein localization in MDA-MB-468 cells. Representative immunofluorescence pictures of RAGE signal (green) in control cells as well as in treated cells with 800 ng/mL recombinant HMGB1 or/and 1 mM metformin (METF) (**A**) and graph from analysis. At least 100 cells per condition were counted (**B**). Representative immunofluorescence pictures of NF-kB signal (green) in control cells as well as in cells treated with 800 ng/mL recombinant HMGB1 or/and 1 mM METF (**A**) and Western blotting results from nuclear extracts (**C**). All data are given as means with SD. Asterisks indicate significant difference ** *p* < 0.01, vs. control. Nuclei are stained with DAPI (blue).

Initial MTT assays determined the IC_50_ concentrations of metformin to be 2.60 mM for the MDA-MB-468 cell line and 8.5 mM for the MDA-MB-231 cell line (Figure S1). All subsequent experiments were performed with IC_25_ metformin concentrations of 1 mM for MDA-MB-468 and 3 mM and MDA-MB-231, respectively, to study the impact on HMGB1/RAGE signaling, EMT, cell motility, and NF-κB nuclear translocation with minimal confounding cytotoxicity. Using IC_25_ ensures that a significant proportion of the cells remain viable, allowing the distinction between metformin’s direct mechanistic effects and general growth inhibition. Higher doses, such as IC_50_, could induce excessive cell death, obscuring pathway-specific effects. To assess the effect of metformin on RAGE receptor stability at the cellular membrane, MDA-MB-468 cells were treated with 800 ng/mL HMGB1 or co-treated with HMGB1 and metformin for 24 h. Immunofluorescence analysis revealed that, under normal conditions, the RAGE signal in MDA-MB-468 cells is low and predominantly distributed throughout the cytoplasm. A similar cytoplasmic distribution was observed following IC_25_ metformin treatment (Figure 1A).

Treatment with HMGB1 alone intensified the RAGE signal, predominantly localizing it at the membrane (Figure 1A,B). However, this membrane localization was markedly reduced when cells were co-treated with HMGB1 and metformin (Figure 1A,B), suggesting that metformin disrupts HMGB1-induced RAGE translocation. To further investigate metformin’s effects, we examined its impact on downstream signaling pathways, focusing on the activation of NF-κB, a key target of RAGE signaling. HMGB1 treatment promoted pronounced nuclear translocation of NF-κB p65 (Figure 1A), indicating activation. The nuclear extracts from cells treated with HMGB1 were more enriched with NF-kB p65 protein in comparison with the control (Figure 1C). In contrast, this translocation was absent in cells co-treated with HMGB1 and metformin (Figure 1A,C). Although the effect was less pronounced, we also observed a similar effect in MDA-MB-231 cells (Figure S2A)

### 3.2. Metformin Prevents HMGB1-Induced Matrix Metalloproteinase-2 Upregulation as Well as EMT in TNBC

**Figure 2:**
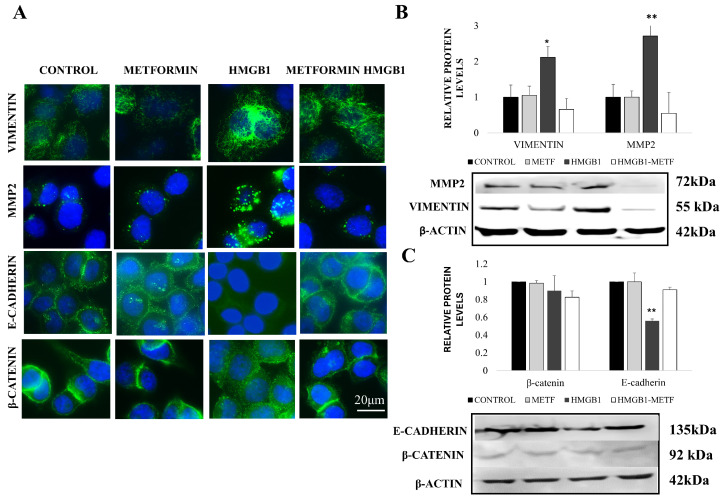
Effect of metformin on HMGB1-induced expression of MMP2 and EMT markers. (**A**) Immunolocalization of EMT markers E-cadherin, vimentin, and β-catenin as well as MMP2 protein (green fluorescence). Nuclei are stained with DAPI (blue). (**B**) Relative protein levels of MMP2 and vimentin in control MDA-MB-468 cells and cells treated with HMGB1 and/or 1 mM metformin (METF). (**C**) Relative protein levels of E-cadherin and β-catenin in control MDA-MB-468 cells and cells treated with HMGB1 and/or 1 mM metformin. Data represent mean ± SD (*n* = 3); * *p* < 0.05, ** *p* < 0.01.

Since NF-κB activation is closely associated with tumor invasion and metastasis, primarily through the regulation of matrix metalloproteinase-2 (MMP-2) [41], we subsequently examined the MMP-2 signal in MDA-MB-468 cells treated with HMGB1. In control cells, MMP-2 was localized to a few dot-like structures in the cytoplasm. Upon treatment with 800 ng/mL HMGB1 for 24 h, the number of these granules (likely secretory vesicles/endosomes) increased (Figure 2A and Figure S3A).

However, this increase was abolished entirely in cells co-treated with 1 mM metformin, suggesting that metformin disrupts HMGB1-induced MMP-2 upregulation (Figure 2A,B and Figure S3A). These results were also confirmed by Western blotting (Figure 2B). Notably, metformin alone did not affect the distribution of either NF-κB p65 or MMP-2 signals at the IC_25_ concentration (Figure 1, Figure 2A,B and Figure S3A). Similar results for MMP2 levels in treated cells were obtained with MDA MB 231 cells (Figures S3B and S2A,B).

We also examined the effect of extracellular HMGB1 on initiating the EMT process in TNBC cells and the influence of metformin on this process. We quantified the protein levels to assess EMT and analyzed the localization of key EMT markers, specifically E-cadherin, β-catenin, and vimentin (Figure 2). During the epithelial-to-mesenchymal transition (EMT), E-cadherin expression decreases, leading to the loss of cell–cell adhesion and increased cell motility. In contrast, Vimentin levels increase, and β-catenin translocates to the nucleus, promoting cytoskeletal reorganization and enhancing the migratory and invasive properties of the cells. The MDA-MB-468 cells were treated with 800 ng/mL HMGB1 and/or metformin. Immunofluorescence and Western blot analyses revealed significant changes in the expression and localization of these EMT markers depending on the treatment. In the control cells, E-cadherin expression was strong and vimentin levels were low, as confirmed by Western blotting (Figure 2A,B).

However, treatment with 800 ng/mL recombinant HMGB1 led to a marked decrease in E-cadherin staining and increased vimentin expression (Figure 2A). This was further validated by Western blot analysis, which showed a reduction in E-cadherin levels and a corresponding increase in vimentin production (Figure 2B). Upon treatment, the levels of vimentin were changed similarly in MDA MB231 cells too (Figure S2A,C). However, β-catenin protein levels remained unchanged after HMGB1 treatment (Figure 2B); the protein exhibited a loss of membrane localization (Figure 2A). It was translocated to the cytoplasm (Figure 2A). These findings support the involvement of HMGB1 in the initiation and progression of the EMT process. Treatment with metformin reversed these changes, leading to an increase in E-cadherin expression, a decrease in vimentin levels, and the inhibition of β-catenin translocation to the cytosol (Figure 2A,B). These results suggest that metformin can counteract HMGB1-induced EMT, potentially inhibiting the migratory and invasive capacities of TNBC cells.

### 3.3. Metformin Inhibits HMGB1-Induced Cell Motility and Proliferation

**Figure 3:**
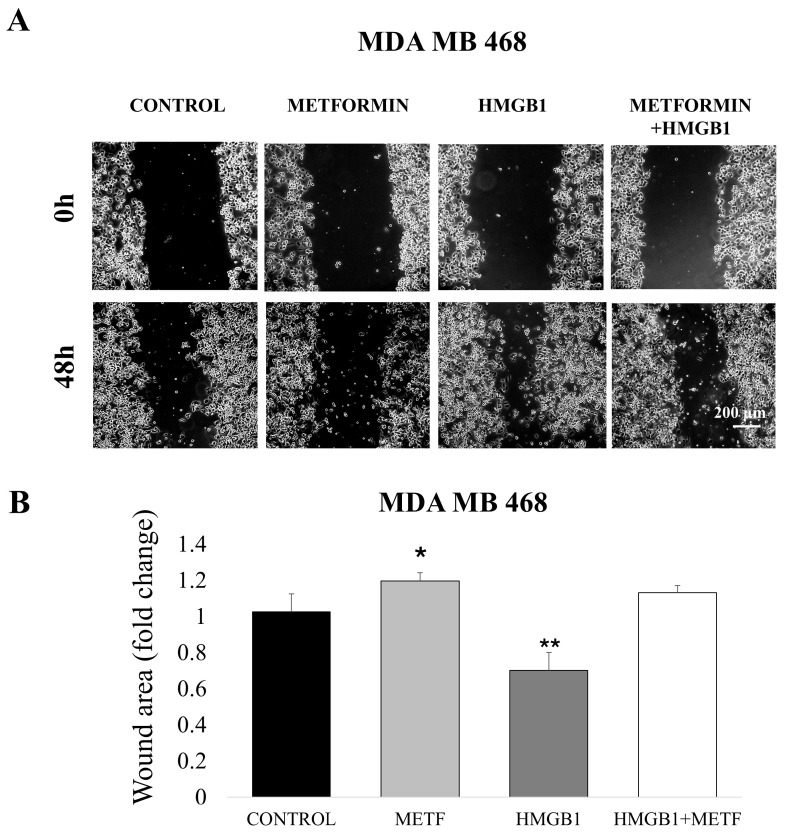
Effect of metformin (METF) on HMGB1-induced migration of breast cancer cells. (**A**) Representative pictures of wound closure in MDA-MB-468 cells treated with 800 ng/mL HMGB1 and/or 1 mM metformin at 0 h and 48 h. (**B**) Quantification of wound healing. Data represent means ± SD; * *p* < 0.05 and ** *p* < 0.01 vs. control.

**Figure 4:**
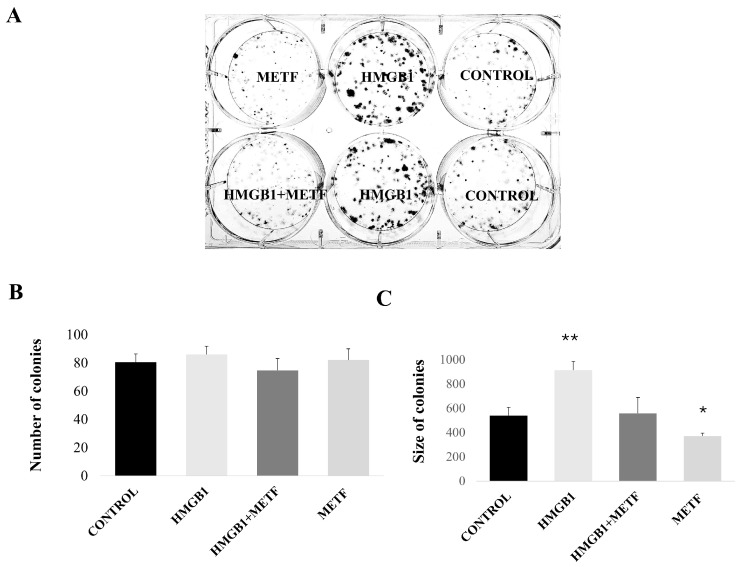
Effect of metformin (METF) on HMGB1-induced proliferation of breast cancer cells. (**A**) Representative images of clonogenic assay with MDA-MB-231 cells. (**B**) Number of colonies. (**C**) Size of colonies. Data represent mean ± SD (*n* = 3); * *p* < 0.05, ** *p* < 0.01, vs. control.

The HMGB1/RAGE pathway is critical in regulating gene expression associated with cell migration and survival. Since metformin inhibits HMGB1-induced NF-κB activation and EMT, we sought to assess its impact on HMGB1-induced cell proliferation and migration. To investigate cell migration, we conducted a wound-healing assay. MDA-MB-468 cells were treated with 800 ng/mL HMGB1 and 1 mM metformin, either alone or in combination, for 48 h. The wound area was quantified using ImageJ software. The results showed that HMGB1-treated cells exhibited a decrease in wound area after 48 h, indicative of enhanced migration. However, cells treated with 1 mM metformin displayed a markedly reduced wound-healing capacity, even when combined with recombinant HMGB1, suggesting that metformin significantly inhibits cell motility (Figure 3). Similar results were also observed with the MDA MB 231 cell line (Figure S4). The HMGB1/RAGE axis has also promoted tumor cell proliferation in certain cancers [42]. To explore this further, we utilized a clonogenic assay to assess the proliferative capacity of MDA-MB-231 cells and the effect of metformin on HMGB1-induced proliferation (Figure 4). Due to the low cell density required for this assay, we employed a metformin concentration of 1.7 mM (an IC_25_ concentration tailored to the cell number).

Cells treated with 800 ng/mL recombinant HMGB1 formed a similar number of colonies to the control group; however, the colonies were significantly larger (Figure 4).

Co-treatment with 1.7 mM metformin resulted in a reduction in colony size comparable to the control, indicating that metformin effectively counteracts HMGB1-driven cell proliferation in breast cancer cells. These findings suggest that metformin impairs HMGB1-induced cell migration and blocks HMGB1-mediated proliferative signaling in breast cancer.

**Figure 5:**
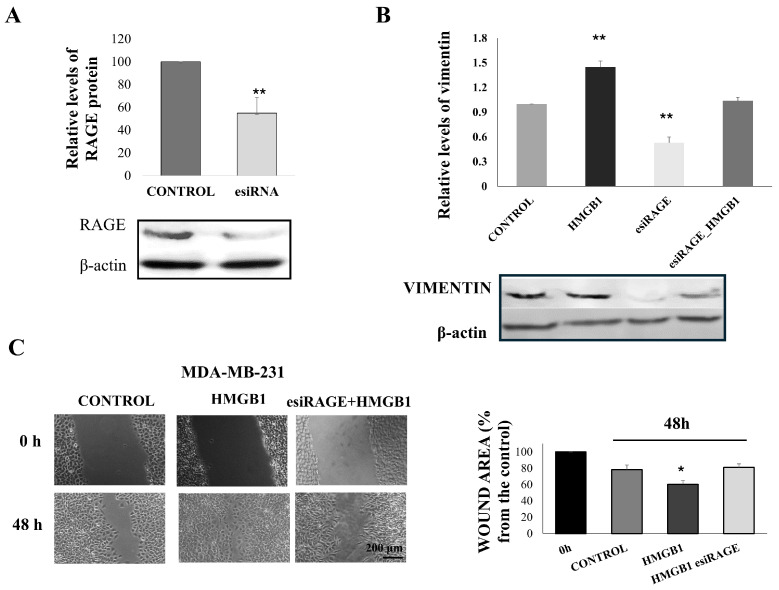
Migration and EMT of MDA-MB-231 cells induced by HMGB1 depends on RAGE receptor levels. (**A**) RAGE protein levels in control and esiRAGE-treated cells. (**B**) Relative levels of vimentin in control or RAGE-silenced cells, treated with HMGB1. Data represent means ± SD (*n* = 3). (**C**) Representative pictures of wound closure in control and RAGE-silenced cells treated with HMGB1 and the quantification of wound healing. Data represent means ± SD; * *p* < 0.05, ** *p* < 0.01.

Furthermore, we confirmed that HMGB1-induced EMT and increased breast cancer cell migration depend on RAGE expression. Targeted knockdown of RAGE using esiRNAs led to a decrease in about half of the RAGE protein levels (Figure 5A).

Vimentin protein levels were compared between the control and RAGE-silenced MDA-MB-231 cells. RAGE silencing led to a decrease in vimentin levels (Figure 5B). Stimulation of the silenced cells with 800 ng/mL HMGB1 increased vimentin protein expression, but this increase was significantly less than in cells with normal RAGE levels (Figure 5B). The stimulatory effect of HMGB1 on cell migration was also abolished in RAGE-silenced cells (Figure 5C).

### 3.4. Metformin Decreases HMGB1 and RAGE Protein Levels in a Concentration-Dependent Manner

**Figure 6:**
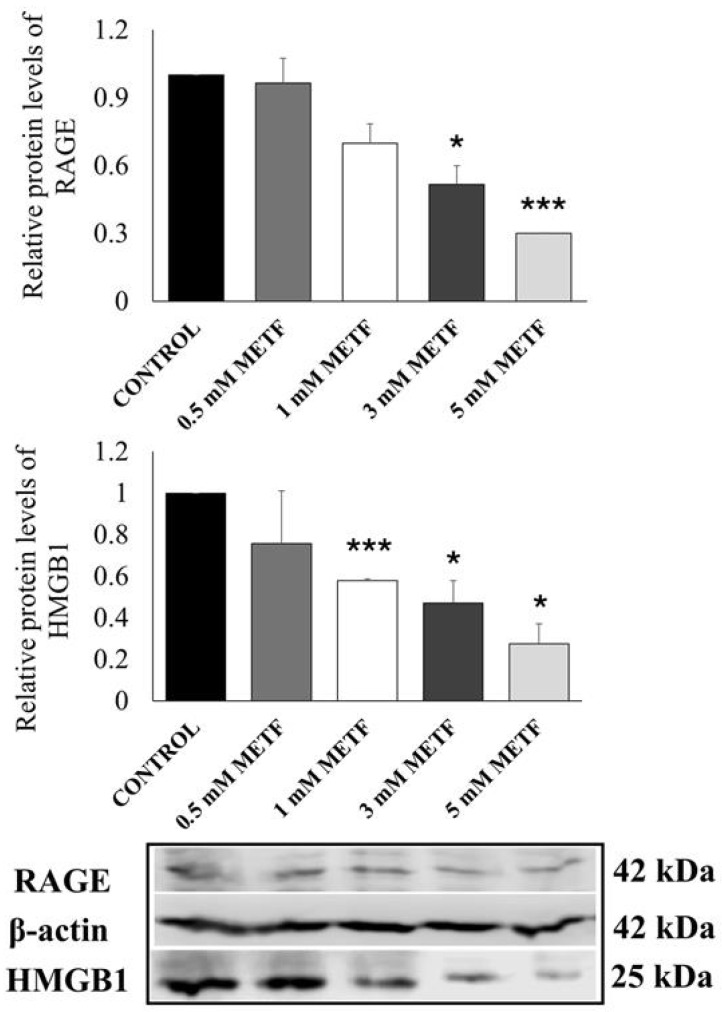
Protein expression levels of HMGB1 and RAGE in MDA-MB-468 cells treated with increasing concentrations of metformin (METF). Data are given as mean and SD. Asterisks indicate a significant difference; * *p* < 0.05, *** *p* < 0.001 vs. control.

RAGE activation by its ligands initiates a positive feedback loop that maintains elevated RAGE expression [43]. Given that metformin can bind to HMGB1 [27] and inhibit RAGE activation, we hypothesized that metformin would subsequently affect RAGE expression levels. Previous studies have shown that metformin inhibits HMGB1 expression in vascular smooth muscle cells [44]. Therefore, we also assessed HMGB1 levels in TNBC cells treated with metformin. MDA-MB-468 cells were exposed to varying metformin concentrations (0–5 mM) for 48 h. Following treatment, the cells were lysed, and the protein levels were analyzed by Western blotting. The results demonstrated that metformin reduces both RAGE and HMGB1 protein levels in a dose-dependent manner (Figure 6).

We also confirmed these results in MDA MB 231 cells (Figure S5).

## 4. Discussion

Metformin, a well-established anti-diabetic medication, has garnered attention for its repurposing in various clinical applications, including its potential as an anticancer agent [45]. Notably, HMGB1 was identified as a novel target for metformin binding through affinity purification with a biotinylated metformin analog. Metformin specifically binds to the C-terminal acidic tail of HMGB1 and has been shown to inhibit HMGB1-induced inflammatory responses both in vitro and in vivo; however, this inhibitory effect is absent when the acidic tail of HMGB1 is lacking [27]. Based on these findings, we hypothesized that metformin’s interaction with HMGB1 would interfere with its binding to the receptor for advanced glycation end products (RAGE), thereby influencing cancer metastasis. In this study, we investigated the effect of metformin on the HMGB1/RAGE signaling axis in TNBC cells. We observed that metformin effectively inhibited cancer cell migration, proliferation, and EMT. Our immunofluorescence analysis revealed that HMGB1 treatment enhances RAGE localization to the cell membrane, a key event in promoting downstream signaling. This membrane localization was disrupted when cells were co-treated with metformin, suggesting that metformin interferes with the HMGB1/RAGE interaction. This disruption was further reflected in the downstream signaling pathways, particularly NF-κB activation. NF-κB, a critical mediator in RAGE signaling, translocates to the nucleus upon activation, regulating the expression of genes involved in tumor invasion and metastasis [46]. In our study, the co-treatment of cells with HMGB1 and metformin resulted in a significant reduction in NF-κB nuclear translocation, indicating that metformin effectively inhibits this pro-metastatic signaling cascade.

**Figure 7:**
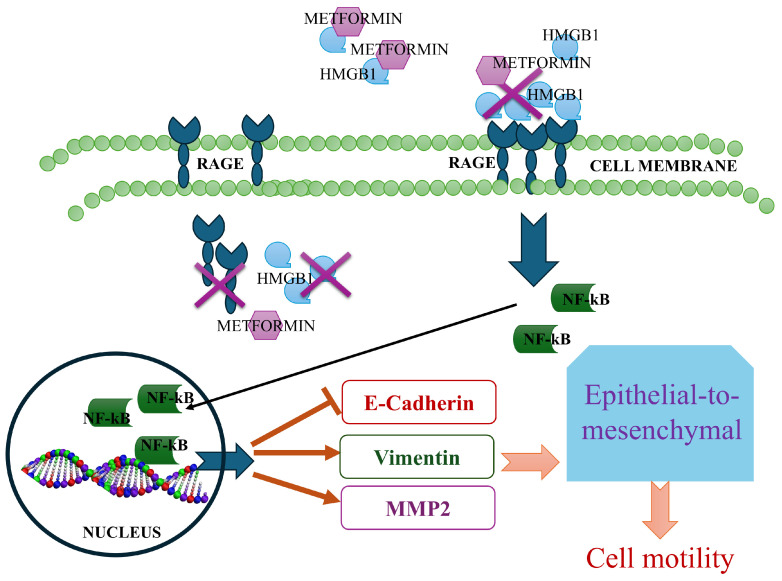
Proposed mechanism of metformin: HMGB1 binding to RAGE stabilizes the receptor on the membrane and activates downstream NF-κB signaling. This leads to p65 translocation into the nucleus, triggering the transcriptional activation of Vimentin and MMP2 while downregulating E-cadherin, thereby inducing the epithelial–mesenchymal transition (EMT) and increasing cell motility. Metformin prevents RAGE stabilization, likely by binding to HMGB1 and blocking its interaction with the receptor (purple ×), thereby inhibiting RAGE downstream signaling. Additionally, metformin suppresses HMGB1 and RAGE expression, disrupting the positive feedback loop that drives cancer aggressiveness.

In addition to its effects on NF-κB, metformin also impacted the expression of matrix metalloproteinase-2 (MMP-2), a protein associated with tumor invasion [47]. Increased expression of MMP-2 in response to HMGB1 has been previously documented in lung cancer cells, and this expression was significantly reduced by NF-κB inhibition [48]. Consistent with these findings, our data showed that metformin diminished HMGB1-induced MMP-2 upregulation, suggesting that metformin’s inhibitory effect extends to critical downstream molecules involved in cancer metastasis (Figure 7).

Furthermore, we observed a dose-dependent decrease in HMGB1 and RAGE protein levels following metformin treatment. This finding is significant as the HMGB1/RAGE axis is known to be upregulated in various cancers, promoting tumor progression through a positive feedback loop where RAGE activation sustains elevated RAGE expression [15,16,17]. By reducing the levels of both HMGB1 and RAGE, metformin disrupts this feedback mechanism, potentially limiting the cell’s ability to maintain an aggressive phenotype. The functional consequences of metformin’s inhibition of the HMGB1/RAGE pathway were demonstrated in our wound-healing and colony formation assays. Metformin significantly reduces cell motility and proliferation, even in the presence of HMGB1, indicating its anti-migratory and anti-proliferative effects. These findings are crucial as they suggest that metformin may impede key processes involved in metastasis, reinforcing its potential as a therapeutic agent for aggressive cancers such as TNBC.

In addition to its anti-migratory and anti-proliferative effects, metformin also demonstrated an ability to reverse the EMT process induced by HMGB1. EMT is a critical step in cancer metastasis, enabling epithelial cells to acquire mesenchymal characteristics, thereby enhancing their migratory and invasive potential. Our results showed that metformin treatment led to an increase in E-cadherin and a decrease in vimentin levels, indicating a reversal of HMGB1-induced EMT. Additionally, metformin stabilized β-catenin at the cell membrane, preventing its translocation to the cytoplasm, which further supports its role in inhibiting EMT and, by extension, metastasis. We also confirmed that HMGB1-induced EMT and the increased migration of breast cancer cells in vitro depend on RAGE expression levels. Lowering RAGE protein levels completely prevented HMGB1-induced cell migration. Interestingly, HMGB1 was still able to induce vimentin expression to some extent even when RAGE levels were low, suggesting that while vimentin expression is primarily regulated by the HMGB1/RAGE pathway, HMGB1 may also influence vimentin through RAGE-independent pathways, such as signaling through TLR4 [49].

Metformin stabilizes GM1-rich lipid rafts in TNBC, modulating EGFR signaling pathways that drive tumor proliferation and survival [50]. While the role of lipid rafts in other diseases, such as antiphospholipid syndrome (APS), has been explored [51], an open question is whether they directly regulate RAGE activity and how metformin’s effects on these membrane domains may influence RAGE-mediated pathways. In this context, it is also important to consider the interplay between HMGB1/RAGE signaling and other inflammatory mechanisms. Interestingly, inhibition of heparanase—an enzyme involved in extracellular matrix degradation and tumor progression—significantly mitigates inflammation-driven thrombosis [52]. Given that heparanase and HMGB1 can both modulate the tumor microenvironment and facilitate metastasis, targeting these parallel pathways may offer synergistic benefits in aggressive cancers such as TNBC.

## 5. Conclusions

Our study demonstrates that metformin exerts multiple inhibitory effects on the HMGB1/RAGE signaling axis in TNBC cells. By disrupting HMGB1/RAGE interactions, inhibiting NF-κB activation, reducing MMP-2 expression, reversing EMT, and lowering HMGB1 and RAGE protein, as metformin is an already FDA-approved drug, it has the potential to be repurposed as an accessible, low-toxicity treatment for TNBC patients with high HMGB1/RAGE activity.

## Data Availability

The datasets used and/or analyzed during the current study are available from the corresponding author upon request.

## Supplementary Materials

The following supporting information can be downloaded at: https://www.mdpi.com/article/10.3390/cells14080590/s1, Figure S1: Effect of metformin on the proliferation of MDA MB 231 and MDA MB 468 cell lines; Figure S2: Immunolocalization of NF-kB p65, vimentin and MMP-2 protein (green fluorescence) in MDA MB 231 cell line; Figure S3: Number of MMP-2 granules in control and cells treated with HMGB1 and/or metformin from MDA MB 468 (A) and MDA MB 231 breast cancer lines (B); Figure S4: The effect of metformin (METF) on HMGB1-induced migration of breast cancer cell line MDA MB 231; Figure S5: Protein expression levels of HMGB1 and RAGE in MDA-MB-231 cells treated with increasing concentration of metformin (METF).
