# Supplementary material for: Metformin Inhibits Cell Motility and Proliferation of Triple-Negative Breast Cancer Cells by Blocking HMGB1/RAGE Signaling
Source: Cells. 2025 Apr 13;14(8):590. doi: 10.3390/cells14080590 (PMC12025684; doi:10.3390/cells14080590)
Supplement: Supplementary file 1 [file cells-14-00590-s001.zip › cells-3562374-supplementary.pdf]

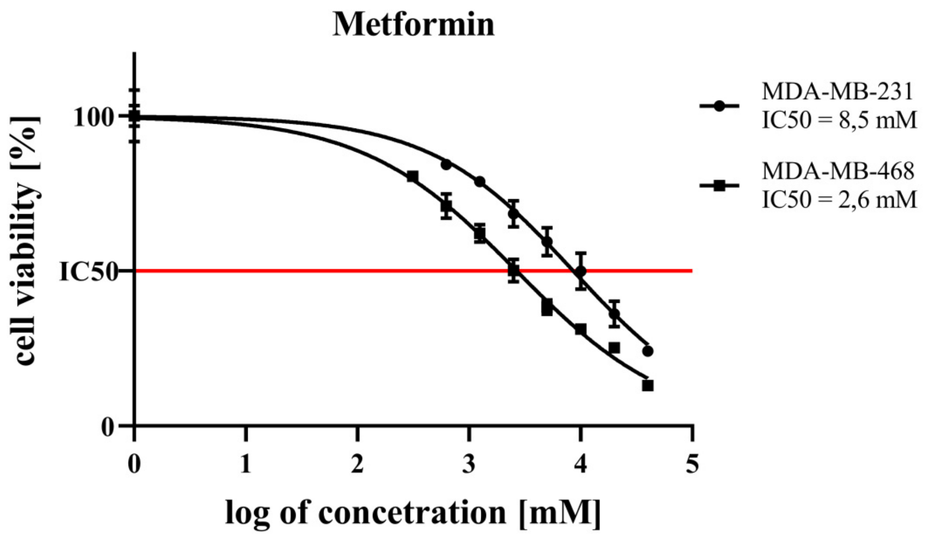

FIGURE S1. Effect of metformin on the proliferation of MDA MB 231 and MDA MB 468 cell lines. Dose-response relationship curves were determined using MTT assay. Data are given as mean and SD.

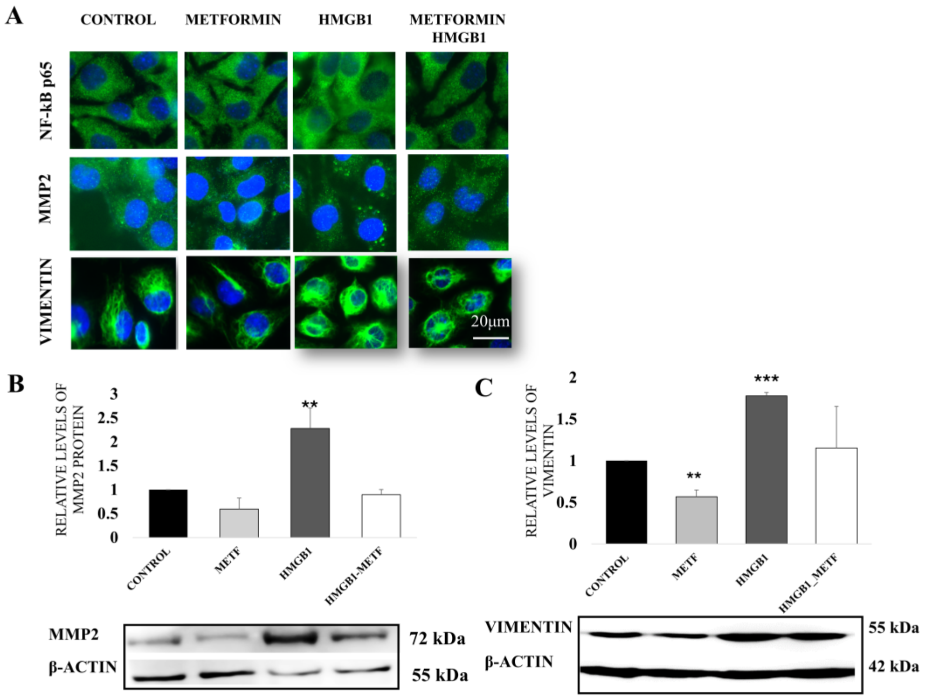

FIGURE S2. Immunolocalization of EMT markers NF-kB p65 and vimentin as well as MMP2 protein (green fluorescence). A: Representative pictures. Nuclei are stained with DAPI (blue). Relative protein levels of MMP2 **(B)** and vimentin **(C)** in control MDA-MB-231 cells and cells treated with HMGB1 and/or 3 mM metformin (METF). Data represents mean ± SD (n = 3), *p≤0.05, **p≤0.01, ***p≤0.001.

A

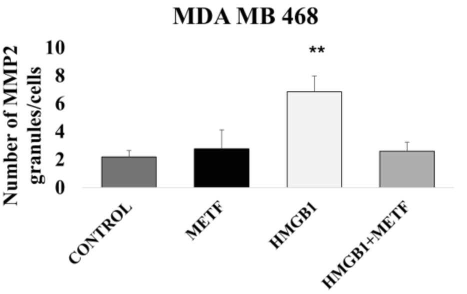

B

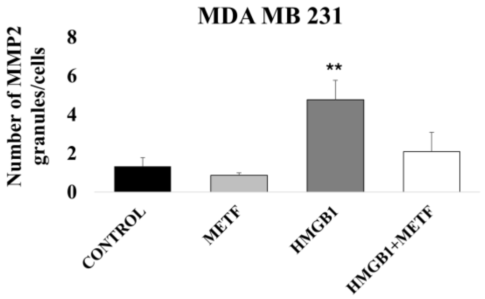

FIGURE S3. Number of MMP2 granules in control and cells treated with HMGB1 and/or metformin from MDA MB 468 (A) and MDA MB 231 breast cancer lines (B). Data represents mean ± SD (n = 3), *p≤0.05, **p≤0.01, ***p≤0.001.

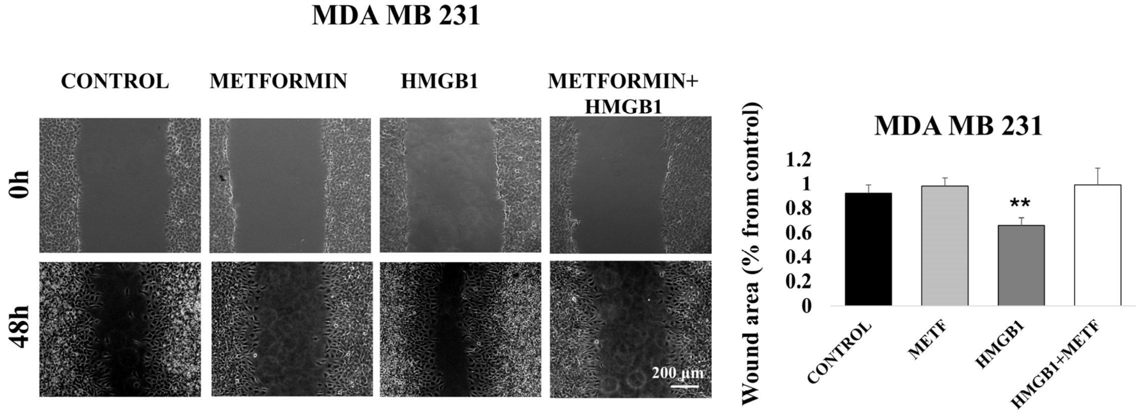

FIGURE S4. The effect of metformin (METF) on HMGB1-induced migration of breast cancer cells. **A:** Representative pictures of wound closure in MDA-MB-231 cells treated with different concentrations HMGB1 and/or 3 mM metformin at 0 h and 48 h. **B:** Quantification of wound healing. Data represents means ± SD, *p ≤ 0.05; **p≤0.01 vs. control.

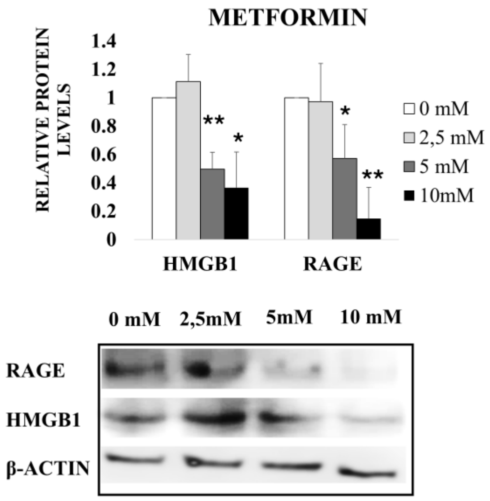

FIGURE S5. Protein expression levels of HMGB1 and RAGE in MDA-MB-231 cells treated with increasing concentration of metformin (METF). Data are given as mean and SD. Asterisks indicate a significant difference *p≤0.05, **p≤0.01, ***p≤0.001 vs. control.
